# Function-Based Diagnosis and Physiotherapeutic Intervention for Shoulder Pain and Scapulothoracic Movement Control Deficits in Volleyball Players: A Case Report

**DOI:** 10.7759/cureus.78952

**Published:** 2025-02-13

**Authors:** Samiya Khan, Kalpana Zutshi, Aafreen LNU, Ashfaque Khan, Abdur Raheem Khan

**Affiliations:** 1 Physiotherapy, Jamia Hamdard University, New Delhi, IND; 2 Faculty of Medicine and Health Sciences, Department of Physiotherapy, Integral University, Lucknow, IND

**Keywords:** biomechanics, functional diagnosis, physiotherapy, shoulder mobility sports injury, sports injury

## Abstract

Overhead athletes require complete, unrestricted arm movements to perform their peak-level sports. As a result, the shoulders must maintain a careful balance between mobility and stability. Volleyball players frequently experience shoulder pain and mobility limitations due to the repetitive overhead motions involved in spiking and serving, which place significant strain on the shoulder joint. This case report examines the functional diagnosis and physiotherapeutic intervention for a 26-year-old male volleyball player suffering from right shoulder pain and limited mobility during overhead activities. The assessment revealed deficiencies in scapulothoracic movement, highlighting the crucial role of the scapular function in shoulder joint mobility. Physiotherapeutic intervention, which focused on restoring scapular function through a six-week conservative physiotherapy intervention, successfully relieved symptoms of shoulder pain and scapulothoracic movement control deficits. These findings underscore the importance of addressing scapular movement in a comprehensive shoulder rehabilitation program, especially for athletes with specialized shoulder requirements, such as volleyball players. The repetitive overhead motions involved in volleyball can lead to a high incidence of shoulder pain and scapulothoracic movement control deficits due to its multifactorial etiology. Function-based diagnosis and physiotherapeutic intervention in athletes emphasize the impact of the condition on sport-specific performance, involving a comprehensive evaluation of biomechanics, range of motion, strength, and scapular mobility and stability. This case study provides valuable insights to guide personalized, movement-based rehabilitation programs for overhead athletes, contributing to the growing body of literature on functional diagnosis and targeted interventions for shoulder disorders in sports persons.

## Introduction

Volleyball is a popular sport that requires repetitive overhead motions, which can lead to a high incidence of shoulder pain and dysfunction among participants. This is due to the physical demands and nature of key volleyball actions, such as spiking, setting, serving, and blocking. As a result, understanding the factors that contribute to these shoulder-related issues in volleyball players is an important area of research and clinical practice [1,2].

Previous research by Kugler et al. was among the first to document the impact of chronic overuse on shoulder girdle mechanics in volleyball players, highlighting asymmetric scapular positioning and limited range of motion in the shoulder girdle on the dominant side among elite athletes. Although scapular dysfunction on the dominant side is now recognized as a common issue in overhead athletes, there has been limited follow-up research specifically examining this phenomenon in the volleyball population since their influential study [3].

Most studies examining shoulder girdle function in volleyball players have emphasized the relevance of the ratio between internal and external rotational isokinetic strength. These studies generally indicate that imbalances in these strength ratios are linked to a history of shoulder injuries in adult volleyball players [4]. 

Functional-based diagnosis in rehabilitation emphasizes identifying movement patterns, biomechanical issues, and neuromuscular problems that contribute to injuries rather than focusing solely on symptoms or anatomical problems [5]. This method assesses how the entire body functions during activities or movements that are relevant to daily life or sports [6]. This case study aimed to assess the functional deficits and movement patterns that contribute to shoulder pain and scapular dyskinesia in volleyball players.

This case study focused on identifying and addressing the underlying biomechanical and neuromuscular impairments that lead to improved shoulder function, reduced pain, and prevention of future injuries. It provides data to guide personalized, movement-based rehabilitation programs for overhead athletes, such as volleyball players. This study contributes to the growing literature on functional diagnosis and targeted interventions for shoulder disorders in sports. Studying the specific movement deficits in this population will enable clinicians to develop more effective, prevention-focused training programs to optimize shoulder health and performance in volleyball players.

## Case presentation

A 26-year-old male student and university-level volleyball player reported a sudden onset of right shoulder pain that occurred two months before the initial visit while playing volleyball and was unable to perform overhead activities. Subsequently, the patient received conservative treatment in the form of ice and sling immobilization. After a few days of immobilization, the patient again started playing volleyball, and during the smashing activity, he experienced pain again. During the time of symptom onset, he recalled going through daily work. The pain progressed throughout the following month, and he began noticing increased pain during overhead activity, activities of daily living (ADL), and at night. No relevant medical or family history was present, and his musculoskeletal system was impaired, as evidenced by his right shoulder pain and difficulty in ADL. The patient was an unmarried, slim, physically active, and non-vegetarian student. Based on observations and patient discussions, the patient’s communication, affect, cognition, and learning were unimpaired.

Clinical findings

The patient’s deficits were categorized using the International Classification of Functioning, Disability, and Health Model [7]. Visual Analog Scale (VAS), goniometric measurements, manual muscle testing (MMT), and Quick DASH were used as outcome measures.

**Figure 1:**
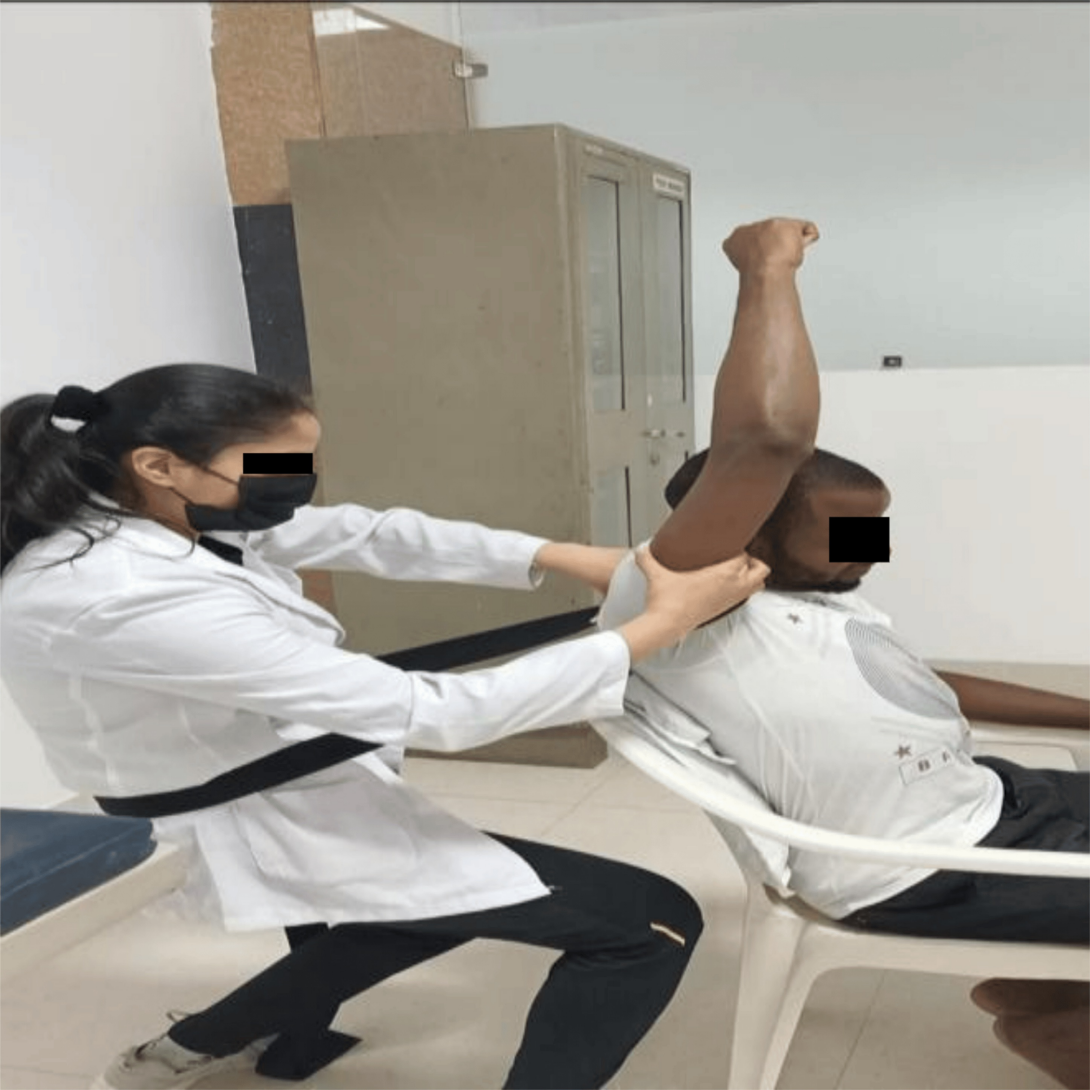
Movement with mobilization at the glenohumeral joint The image is clicked by the author.

**Figure 2:**
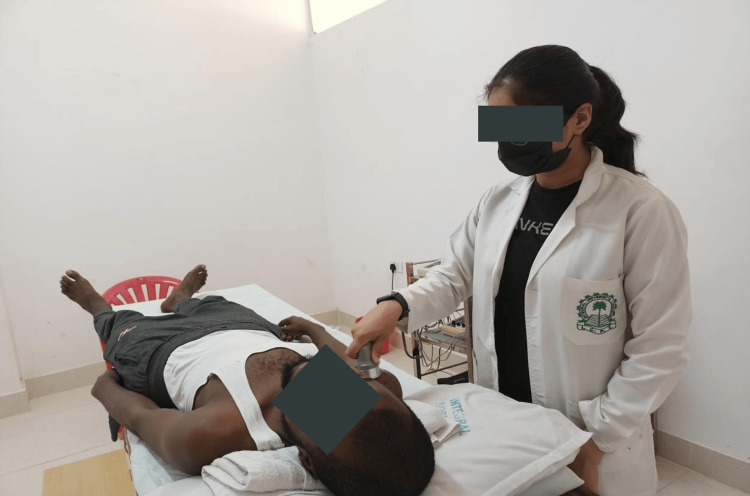
Application of ultrasound therapy at the glenohumeral joint The image is clicked by the author.

The examination involves body functioning and structure examination focusing on posture, pain, range of motion (ROM), sleep, ADLs report, and strength. These are documented in Table 1. Functional status involving functional activity and special tests is listed in Table 2. No imaging study advice was given to the patient because of a function-based diagnosis. Table 3 shows the net improvement in outcomes through therapeutic intervention for body function. Table 4 shows the net improvement in outcomes through therapeutic intervention for activity limitation. Figure 1 shows GH accessory mobilizations, which are techniques used to improve joint mobility, specifically targeting the glenohumeral joint (shoulder joint). Figure 2 shows ultrasound therapy applied to the patient for 7 minutes at 1.5 W/cm².

**Table 1:** Functional activity and special test UE: Upper extremity, DASH: Disabilities of arm, shoulder and hand; DASH-W: Disabilities of arm, shoulder and hand at work.

Measurement category	Test/Measure used	Test/Measure results pre-treatment	Test/Measure results post-treatment
UE functional activity	Quick DASH	45.5%	6.8%
Participation in work duties	Patient report quick DASH-W	52.3%	4.5%
Special test	Hawkins Kennedy/ impingement test	Positive	Negative

**Table 2:** Examination data of body function and structure VAS: Visual analogue scale, ROM: Range of motion, MMT: Manual muscle testing, (+): Present, (-): Absent

Category	Outcome measure	Baseline assessment	After six weeks
Posture	Observation	Scapula elevated, protracted, forward rotated and anterior tipping. Dorsal spine mild posterior convexity present	Improve on visual estimation
Pain	VAS	7	2
ROM	Goniometer	Flexion-170 Extension-50 Abduction-165 External rotation-80	Flexion-175 Extension-55 Abduction-175 External rotation-90
Accessory motion	Inferior and posterior glide	Shoulder joint inferior glide: hypo mobile pain (+) posterior glide: hypo mobile, pain (+)	Shoulder joint inferior glide: normal mobile pain (-) posterior glide: normal mobile, pain (-)
Shoulder and scapular stabilizer strength	MMT	Flexion-5/5 Abduction-4/5 Internal rotation-4/5 External rotation-4/5 Scapular stabilizer-4/5	Flexion-5/5 Abduction-5/5 Internal rotation-5/5 External rotation-5/5 Scapular stabilizer-5/5

**Table 3:** Physiotherapeutic intervention for impaired function and net improvement after six weeks GH, Glenohumeral jointAP: Anterior-posterior, GH: Glenohumeral joint, Stg: Strengthening, MWM: Movement with mobilization, VAS: Visual analogue scale, MMT: Manual muscle testing, PNF: Proprioceptive neuromuscular facilitation.

Body function	Physiotherapeutic intervention	Outcome measure	Net Improvement in outcome
Posture	Home instruction for posture correction	Observation	Improve on visual estimation
Pain	Patient education regarding self-pain management, ultrasound sound therapy- continuous mode, 1.5 Hz for 7 minutes, cold pack 10-20 min after exercises	VAS	5
ROM	GH physiologic mobilization for abduction grade II and III- 2x30 second bouts. Progressed vigorous by increasing grades to III and IV and increasing bouts to 2x60 seconds	Goniometer	Flexion-5 Extension-5 Abduction-10 External rotation-10
Accessory motion	GH accessory mobilization AP and inferior glides. Grade II and III-, 2x30 second bouts. Progressed vigorous by increasing grades to III and IV and increasing bouts to 2x60 seconds MWM for posterior and anterior glide Therapeutic exercises shoulder pulley for abduction 20 rep 2 session	Inferior and posterior glide	Shoulder joint inferior glide: normal mobile pain (-) Posterior glide: normal mobile, pain (-)
Muscle strength	Exercise in prone 5 sec hold, 5 sec relax, 5 rep, 3 sets, 2 sessions. PNF diagonal with TheraBand (Ohio, USA). External rotator stg with TheraBand 5 sec hold, 5 sec relax, 5 rep, 3 sets, 2 sessions. Prone horizontal ab 10 stg exs 5 sec hold, 5 sec relax, 5 rep, 3 set, 2 sessions.	MMT	Flexion-1 Abduction-1 Internal rotation-1 External rotation-1 Scapular stabilizer-1

**Table 4:** Physiotherapeutic intervention for activity limitation and net improvement after six weeks UE: Upper extremity, DASH: Disabilities of arm, shoulder and hand, ADL: Activities of daily living, PNF: Proprioceptive neuromuscular facilitation, VAS: Visual analogue scale, ROM: Range of motion

Activity limitation	Physiotherapeutic intervention	Outcome measure	Net improvement in outcome
Moderately limited function for UE activities	Same plan of care as in Table 3 for pain, ROM, and strength for improvements in shoulder function	Quick DASH	Improve shoulder function by 38%
Decreased ability to perform ADL’s	Functional strengthening performed in clinic 2x/week • Scapular PNF: S/L anterior elevation/posterior depression x5 minutes	VAS	Patient is able to independently perform activities with no pain (0/10). And sports activities with no pain (0/10)
Disrupted sleep	Same plan as pain management. Educated patient on sleep hygiene and pillow positioning	Quick DASH	Pain does not interfere with the patient's sleep, and they sleep well

Outcome measure

In this case study, we used posture, VAS, DASH, and accessory motion; goniometric measurement of ROM and MMT at pre-intervention at 0 days; and measurement of all outcome measures at the end of six weeks. Pre-to-post-intervention (end of six weeks), all outcome measures showed improvements. The pre-to-post-net improvement in various outcome measures was as follows: posture improved on visual estimation, VAS net improvement 5 points, shoulder ROM improvement Flexion-5 and Extension-5 Abduction-10 External rotation-10 (in degrees). Accessory motion and muscle strength (MMT) improved. DASH improves by 38%.

## Discussion

The patient showed a significant decrease in pain, along with notable improvements in range of motion (ROM) and enhanced strength of the shoulder and scapular stabilizer muscles. These advancements have greatly improved the patient’s capacity to perform everyday activities and elevated their overall quality of life. Increased ROM and muscle strength have enabled smoother and pain-free movement, allowing the patient to participate more fully in daily and recreational activities. Consequently, the patient experienced enhanced well-being and functional abilities. This progress highlights the effectiveness of the targeted rehabilitation strategy, which not only alleviates pain but also emphasizes the restoration of functional movement and muscle strength. Consequently, the patient is now better able to handle daily tasks and enjoy an improved quality of life, demonstrating the success of physiotherapeutic intervention.

The rehabilitation program effectively addressed the identified impairments and activity limitations through a function-based multimodal intervention approach. This comprehensive strategy proved crucial for driving the significant improvements observed in patient outcomes. Targeted interventions focused on pain management, restoration of proper shoulder biomechanics, strengthening of the involved musculature, and reintegration of functional tasks.

The findings from this case study are consistent with those reported in similar studies, which have found that a combined approach of manual therapy techniques, therapeutic exercises, and patient education can lead to significant improvements in clinical outcomes for patients with shoulder pathologies such as adhesive capsulitis [8]. One study indicated that exercise, joint mobilization, and laser therapy are beneficial in reducing pain and enhancing function in individuals with shoulder impingement syndrome and that joint mobilization combined with other interventions can be more effective in treating primary shoulder impingement [9].

This study has several limitations. As a single case study, the findings are based on only one volleyball player, which restricts the ability to generalize the results to a broader population or other athletes. The findings of this case study can also be explained by alternative factors beyond rehabilitation interventions. For instance, the patient’s own natural healing processes, changes in their daily activities or lifestyle, or even a placebo effect from the treatments could have contributed to the observed improvements. The focus on a single type of intervention, without comparison with other methods, further constrains the study's applicability. Finally, the short-term nature of follow-up does not provide insight into the long-term sustainability of the improvements.

Future research on this case study in randomized control trials could take several important directions. Expanding the study sample to include a larger and more diverse population of volleyball players would enhance the generalizability of our findings. Implementing a control or comparison group would provide a clearer understanding of the specific effects of each intervention in the rehabilitation program. Additionally, exploring a range of physiotherapeutic methods, both individually and in combination, could offer valuable insights into the most effective treatment strategies for addressing shoulder pain and functional deficits in this athlete population. Long-term follow-up studies are also needed to assess the sustainability of the improvements observed and the impact on patients' overall athletic performance and quality of life.

## Conclusions

The case study provides valuable insights into the effectiveness of a targeted rehabilitation approach for this population. The study demonstrated promising results in a single volleyball player, where a six-week protocol involving ultrasound, mobilization, and scapular and shoulder strengthening exercises led to significant improvements in muscle strength, a notable reduction in pain, and an enhancement in quality of life and the ability to perform daily activities. These findings suggest that this comprehensive, multimodal intervention has the potential to effectively address shoulder pain and functional deficits in volleyball players. While further research is needed to confirm these outcomes in a broader population, this case study represents an important step in developing evidence-based rehabilitation strategies for this athlete group, which could have meaningful implications for their overall athletic performance and quality of life.
